# Ramucirumab and Docetaxel in Patients with Metastatic Urothelial Carcinoma Harboring Fibroblast Growth Factor Receptor Alterations: A Case Series and Literature Review

**DOI:** 10.4103/JIPO.JIPO_22_19

**Published:** 2020-01-07

**Authors:** Katherine Emilie Rhoades Smith, Emilie Elise Hitron, Greta A Russler, Deborah A Baumgarten, Mehmet Asim Bilen

**Affiliations:** 1 Department of Medicine, Emory University School of Medicine, Atlanta, GA, USA; 2 Department of Hematology and Medical Oncology, Winship Cancer Institute of Emory University, Atlanta, GA, USA; 3 Department of Radiology, Emory University, Atlanta, Georgia, USA

**Keywords:** Bladder cancer, disease progression, fibroblast growth factor, partial response, vascular endothelial growth factor

## Abstract

Metastatic urothelial carcinoma (mUC) has a poor prognosis with a 5-year survival probability of 4.8%. The mainstay of first-line treatment is platinum-based chemotherapy. Second-line therapy involves immune checkpoint inhibitors or a fibroblast growth factor receptor (FGFR) inhibitor, erdafitinib, for patients harboring selected *FGFR* alterations. Several additional agents are under development for the treatment of mUC. Recent studies demonstrate that ramucirumab and docetaxel have clinical activity in mUC. We report two patients with metastatic upper tract urothelial cancer (mUTUC) with *FGFR* alterations who were heavily pretreated with FGFR inhibitors that later showed response to ramucirumab and docetaxel. Preclinical studies indicate that FGF and VEGF pathways work synergistically, which could explain the observations in our patients. Our findings may represent another treatment option for patients with mUC and *FGFR* alterations who have progressed on multiple lines of therapy.

## Introduction

Urothelial carcinoma (UC) is a common malignancy globally with 429,000 new cases and 165,000 deaths a year.[1] In the USA, there was an estimated 81,190 new cases and 17,240 deaths in 2018.[2] Since 1975, there has been relatively little change in the number of new cases, 5-year survival probability, and deaths. Based on Surveillance, Epidemiology, and End Results data from 2008 to 2014, the 5-year survival was 77% for all stages of UC, 68% for localized disease, and 4.8% for metastatic disease.[3]

The first-line treatment for metastatic UC (mUC) is platinum based followed by immune checkpoint inhibitors.[4–6] Recently, a fibroblast growth factor receptor (FGFR) inhibitor, erdafitinib, gained Food and Drug Administration (FDA) approval for mUC with *FGFR2* or *FGFR3* alterations after first-line platinum-based chemotherapy.[7–9] Beyond this, there are many clinical trials underway for targeted therapies. One focus is additional FGFR inhibitors because *FGFR* is frequently altered in mUC with *FGFR3* mutations found in 21% of patients.[10–15] Another recent finding involves ramucirumab, an anti-vascular endothelial growth factor (VEGF) antibody, which is associated with improved progression-free survival (PFS) and objective responses when used with docetaxel.[16–18] However, there are no studies evaluating the efficacy of ramucirumab and docetaxel in relation to genetic alterations and how to best sequence different therapies.

Our case series is the first, to our knowledge, of patients with metastatic upper tract urothelial cancer (mUTUC) and *FGFR* alterations who had clinical benefit to ramucirumab and docetaxel after progression on multiple therapies, including an FGFR inhibitor.

## Case Reports

### Case 1

Case 1 is a 64-year-old male diagnosed with mUTUC of the right renal pelvis in April 2016. He initially underwent neoadjuvant dose-dense methotrexate, vinblastine, doxorubicin, cisplatin before a right nephroureterectomy. The patient developed retroperitoneal lymphadenopathy after surgery, so began second-line therapy with atezolizumab. Progressive disease was noted after five cycles, so he was referred to the Winship Cancer Institute for participation in a clinical trial. Next-generation sequencing testing revealed an *FGFR2* amplification [Table 1], so the patient received an FGFR inhibitor on a clinical trial until disease progression.

**Table 1:** Genomic alterations in case 1 and case 2

**Case 1**		**Case 2**	
	
**Gene**	**Alteration**	**Gene**	**Alteration**
*FGFR2*	amplification	*FGFR3*	*S249C*
*CDK4*	*R24S*	*EP300*	*S531*
*MDM2*	Amplification	*ARID1A*	*Q1573*
		*TSC1*	A173fs*37
		*TERT*	Promoter - 124C >T
		*CDKNN2B*	Loss
		*CDKN2A*	Loss

Case 1 has an *FGFR2* amplification and case 2 has an *FGFR3* alteration, *S249C*. A173fs*37 is the reported alteration in the gene, *TSC*, which is a base substitution in the promoter region. FGFR: Fibroblast growth factor receptor

**Figure 1:**
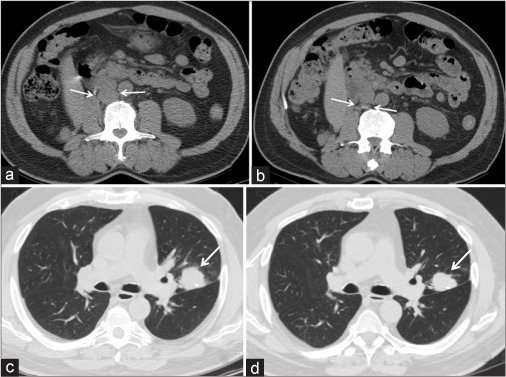
Axial noncontrast computed tomography showing baseline and best response of case 1 (a and b) and case 2 (c and d). (a) Case 1 – enlarged retrocaval node (arrows) (August 14, 2017). (b) Case 1 – significant decrease in node size (arrows) (October 25, 2017). (c) Case 2 – left upper lobe pulmonary mass (arrow) (February 16, 2018). (d) Case 2 – decrease in mass size (arrow) (May 14, 2018)

In August 2017, he was started on ramucirumab (10 mg/kg) and docetaxel (75 mg/m^2^). After three cycles, computed tomography (CT) abdomen/pelvis in October 2017 showed partial response per RECIST v1.1 [Figure 1a and b] with a decrease of 40%. Magnetic resonance imaging in August 2018 showed stable disease with unchanged retroperitoneal lymphadenopathy. The patient experienced toxicity from docetaxel, including nail changes and eye tearing, which prompted a dose reduction from 75 mg/m^2^ to 60 mg/m^2^ in June 2018. In October 2018, he was still alive and well after a total of 17 cycles without disease progression.

### Case 2

Case 2 is a 59-year-old male diagnosed with mUTUC of the left renal pelvis in October 2014. The patient completed neoadjuvant gemcitabine and cisplatin in January 2015 and then had a left nephroureterectomy. In April 2016, imaging revealed new pulmonary nodules. The patient underwent a video-assisted thorascopic surgery (VATS) procedure with wedge resection of the left upper lobe in May 2016 and the right upper lobe in July 2016. In June 2016, the patient had cystourethroscopy with transurethral resection of the bladder neck, limited transurethral resection of the prostate, and mitomycin-C chemotherapy. Disease recurrence was detected in December 2016, so he began atezolizumab. In March 2017, the patient changed to nivolumab due to disease progression at the site of previous wedge resection. In June 2017, he again had disease progression prompting next-generation sequencing testing, which showed *FGFR3* alteration, *S249C* [Table 1]. He received FGFR inhibitor treatment via clinical trial, which was stopped after disease progression.

In March 2018, he began treatment with ramucirumab (10 mg/kg) with docetaxel (75 mg/m^2^). After three cycles, imaging showed an interval decrease in bilateral lung soft-tissue nodularity [Figure 1c and d]. Although the patient achieved stable disease per RECIST v1.1, there was a 27% decrease overall. The patient continued with this treatment for eight cycles. Side effects included fatigue, edema, hiccups, mucositis, hair loss, and joint aches. In August 2018, imaging showed stable disease in the chest, but found a new liver lesion. He underwent ablation of the liver lesions in September 2018. Biopsy confirmed metastatic urothelial cancer, but there was not enough tissue for further genomic testing. Although chest CT remained stable, subsequent imaging showed further progression in the liver and bones, so the patient discontinued treatment in October 2018.

## Discussion

In UC, several alterations in FGFR have been reported, indicating that FGFR inhibitors are a potential approach to individualize therapy.[10–15] In this case series, we reported two patients with mUTUC that carried an *FGFR* alteration and progressed after receiving FGFR inhibitor treatment that later showed clinical benefit to ramucirumab and docetaxel.

The FGF signaling pathway is involved in many processes, including tissue regeneration and angiogenesis.[19] Alterations in the FGF pathway occur in multiple cancers leading to proliferation, resistance to treatment, and neoangiogenesis.[19,20] Due to known *FGFR* alterations in UC, targeted drugs are under investigation. A pan-FGFR inhibitor was found to have a disease control rate of 62.4% and a response rate of 25.4% in patients with mUC who carry *FGFR* alterations.[12–14] Erdafitinib, an FGFR inhibitor specific to FGFR 1–4, now has FDA approval for mUC with *FGFR3* or *FGFR2* alterations.[7–9] Approval was based on Phase 2 trial from Loriot et al., showing an overall response rate (ORR) of 40%. Patients with *FGFR* mutations had better responses with ORR of 49% compared to 16% for patients with *FGFR* fusions.[8] Therefore, despite new innovations in FGFR-targeted therapy, patients need additional options to address various *FGFR* alterations and acquired resistance to FGFR inhibitors.[15,21]

With regard to VEGF therapies in mUC, a Phase II trial found that ramucirumab with docetaxel improves median PFS from 2.8 to 5.4 months compared to docetaxel alone in mUC.[16] A Phase III trial again showed that ramucirumab was associated with an improved PFS of 4.07 months compared to 2.76 months with docetaxel and placebo. There was an objective response of 24.5% in ramucirumab group and 14% in the placebo group.[17,18] Given these data, ramucirumab with docetaxel was used in our patients as a salvage therapy after progressing on an FGFR inhibitor. Case 1 continues to do well nearly 2 years after starting ramucirumab with docetaxel, and Case 2 was clinically stable for 6 months.

Preclinical studies indicate that FGF works synergistically with VEGF and platelet-derived growth factor pathways, which could account for the observations in our patients.[15,21–27] Several mouse model studies found that FGF2 and VEGF promote angiogenesis and lymphangiogenesis, thus facilitating metastasis.[22,23,25,26] In addition, VEGF monoclonal antibodies inhibit FGF-2-induced endothelial cell proliferation and vascularization.[21] Although these data are preclinical, the evidence cited provides a potential mechanism to support the responses we observed in our patients with *FGFR* alterations that responded to anti-VEGF therapy. The difference in responses seen in our patients may be due to variations in the specific FGFR alterations.

## Conclusions

Our case series demonstrates that ramucirumab with docetaxel may benefit patients with *FGFR* alterations who were previously treated with FGFR inhibitors, potentially due to the interactions between FGF and VEGF as demonstrated in preclinical studies. There was a difference in response to ramucirumab with docetaxel in our patients, which could be related to the underlying biology in addition to variations in *FGFR* alterations. Our findings are limited due to a small sample size and lack of correlative studies. Further validation in a larger database or prospective trial is needed.

### Declaration of patient consent

The authors certify that they have obtained all appropriate patient consent forms. In the form the patient(s) has/have given his/her/their consent for his/her/their images and other clinical information to be reported in the journal. The patients understand that their names and initials will not be published and due efforts will be made to conceal their identity, but anonymity cannot be guaranteed.
